# Costunolide Influences Germ Tube Orientation in Sunflower Broomrape – A First Step Toward Understanding Chemotropism

**DOI:** 10.3389/fpls.2021.699068

**Published:** 2021-08-13

**Authors:** Anna Krupp, Barbara Bertsch, Otmar Spring

**Affiliations:** Department of Biochemistry of Plant Secondary Metabolism (190b), Institute of Biology, University of Hohenheim, Stuttgart, Germany

**Keywords:** *Orobanche cumana*, *Helianthus annuus*, plant growth, parasitic plant, seed germination, sesquiterpene lactones

## Abstract

*Orobanche cumana* WALLR. is a host-specific root parasite of cultivated sunflowers with increasing economic importance in Europe, North Africa, and parts of Asia. While sesquiterpene lactones (STLs) released from sunflower roots were identified as natural germination stimulants of *O. cumana* seeds in the soil, the chemical nature of the signals guiding the emerging germ tube toward the host root has remained unknown hitherto. Thus, we designed a bioassay that allowed the observation of broomrape germination and subsequent germ tube development in the presence of substances with putative chemotropic activity. Root exudates and sunflower oil extracts, both containing STLs in micromolar concentrations, caused the positive chemotropic orientation of germ tubes. A similar positive chemotropic effect was achieved with costunolide, one of the four STLs of sunflower present in the exudate and oil extracts. In contrast, GR24, a synthetic strigolactone (SL) with germination-inducing activity on *O. cumana* seeds, showed no effect on the germ tube orientation. The effect of costunolide was concentration-dependent and within the range of its natural micromolar occurrence in roots. We assume that an STL gradient is responsible for the stronger inhibition of elongation growth on the host-facing flank of the germ tube compared with the far side flank. This would confer a double role of STLs from sunflower root exudates in the sunflower–broomrape interaction, namely, as germination stimulants and as chemotropic signals.

## Introduction

Chemical signals are decisive factors for the survival of parasitic plants. Thus, particular terpenoids such as strigolactones (SLs) and sesquiterpene lactones (STLs) released from the hosts have been found to stimulate germination of root parasites from the Orobanchaceae family and secure host specificity (Yoneyama et al., 2013), but germination is only the first step in the life cycle, waking up the parasite by breaking its physiological dormancy. Afterward, the parasite has a very short period of time and limited energy to reach the host root surface by actively growing in the right direction. This process, named chemotropism, is apparently guided by host-derived metabolic signals and is not well understood up to date. The shoot parasite dodder (*Cuscuta pentagona* Engelm.), for example, “smells” its host, as its growth is guided by volatile compounds such as α-pinene (Runyon et al., 2006). The chemotropism involved in root parasitic interactions, which takes place in the chemically complex soil matrix, is even harder to study. While germination of root parasites has extensively been investigated especially in agro-economically relevant species such as *Striga* spp. (Cook et al., 1966, 1972; Siame et al., 1993), *Phelipanche ramosa* (L.) POMEL (Auger et al., 2012), or the sunflower broomrape *Orobanche cumana* WALLR. (Joel et al., 2011; Raupp and Spring, 2013; Ueno et al., 2014), mechanisms directing the parasite germ tube to the surface of the host root are still poorly understood. Early studies by Pearson (1913) and Saunders (1933) suggested that a chemotropic signal is involved in the growth of *Striga* germ tubes. This was supported by Williams (1961) and illustrated by Yoshida and Shirasu (2009). For other genera of *Orobanchaceae*, a putative chemotropic reaction was observed in *Alectra vogelii* Benth. (Botha, 1948; Visser et al., 1977) and *Orobanche crenata* Forssk. (Whitney and Carsten, 1981; Aber and Sallé, 1982), but the chemical nature of the signaling compounds was not unraveled.

**FIGURE 1:**
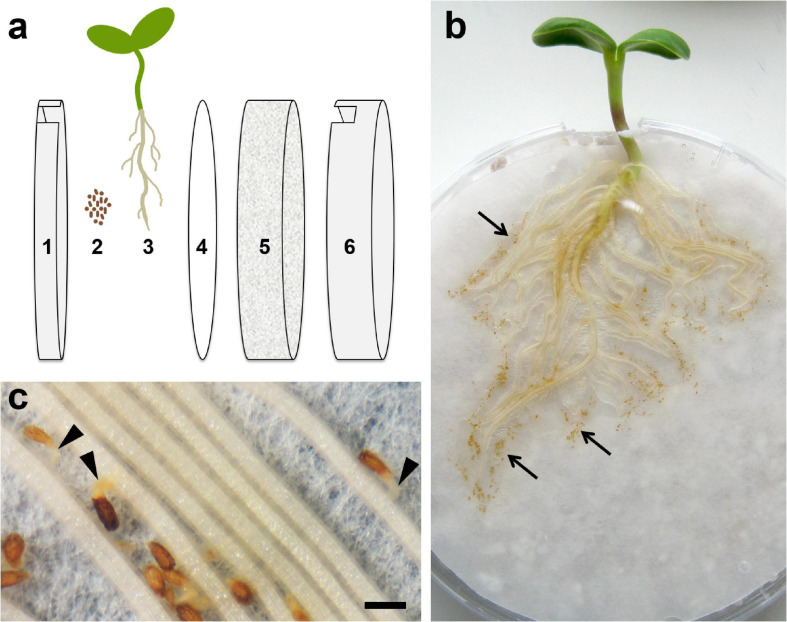
Root chamber (rhizotron) cultivation system. **(a)** Technical setup: 1 – lid of Petri dish, 2 – surface-sterilized seeds of *O. cumana*, 3 – sunflower plant, 4 – filter paper, 5 – perlite, and 6 – bottom of Petri dish. **(b)** Root chamber with a 12-day-old sunflower plant and seeds of *O. cumana* (brownish points, *arrows*) placed near the sunflower roots. **(c)** Germinated seeds of *O. cumana* with germ tubes (*arrowheads*) growing toward the host root. Scale bar = 500 μm.

Chemotropic signal candidates for root parasites have to fulfill a series of specific features. (1) The signal compound must be synthesized and released by the host in a stage susceptible for the attack and suitable for the development of the parasite. (2) The compound has to be stable enough and transportable in soil to reach the parasite. (3) It must show bioactivity related to plant growth processes. STLs of sunflower (*Helianthus annuus* L.) have the potential to fulfill these requirements. They are produced in the early stages of seedling development (Spring et al., 2020), are released with root exudates, effectively induce germination of *O. cumana* (Joel et al., 2011; Raupp and Spring, 2013), inhibit auxin-induced elongation growth (Spring and Hager, 1982), impede polar auxin transport in plants (Ueda et al., 2013), and induce the phototropic (and possibly gravitropic) curvature of sunflower hypocotyls (Yokotani-Tomita et al., 1999; Spring et al., 2020). The four sunflower STLs, dehydrocostus lactone, costunolide, 8-epixanthatin, and tomentosin, present in leaves, stems, and roots of seedlings as well as in sunflower seeds and oil (Spring et al., 2020; Spring, 2021) not only induce the germination of *O. cumana* but simultaneously have the potential to trigger the host-directed growth of the parasitic germ tube observable in rhizotron experiments (Figure 1). It was the aim of this study to test this assumption by establishing a bioassay suitable for observing the germination of seeds and visualizing germ tube development at different distances from the source of target compounds. The results of such bioassays with sunflower root exudates and pure reference compounds were expected to provide conclusive results on whether STLs could be the responsible chemotropic signal for germ tube orientation in the sunflower–broomrape interaction.

## Materials and Methods

### Plant Material

Sunflower (*H. annuus* line HA300) plants were grown hydroponically in 1-L plastic containers for a root exudate collection as described by Raupp and Spring (2013) or co-cultivated with *O. cumana* seeds in a root chamber system to follow germination and germ tube orientation (Figure 1). Seeds of *O. cumana* pathotype G (collected in the Rostov region of Russia in 2012) were kindly provided by T. Antonova and S. Guchetl (All-Russia Research Institute of Oil Crops by the name of V.S. Pustovoit, Laboratory of Immunity and Molecular Marking).

### Seed Treatment for Germination

The seeds of *O. cumana* were surface-sterilized with 70% ethanol for 1 min, 3.6% sodium hypochlorite solution in 1% Tween 80 for 3 min and 30 s in a supersonic bath, followed by 0.01 M hydrochloric acid for 10 min. After each step, the seeds were rinsed thoroughly with deionized water. The seeds were spread on a moist, heat-sterilized Whatman filter (diameter 1.15 cm). For seed conditioning, these filter discs were kept on a moist filter paper in Petri dishes at 18°C in darkness for at least 1 week. The ability to germinate was tested by placing the conditioned seeds in small Petri dishes (diameter 3.8 cm) on Whatman filter discs containing 20 μl of germination stimulant (GR24 1 ppm = 3.3 μM; costunolide 1 μM; sunflower root exudate). The filter paper at the bottom of each dish was moistened with 65 μl ddH_2_O. Each Petri dish was sealed with Parafilm^®^M (Amcor, Zurich, Switzerland) and wrapped in aluminum foil to avoid illumination. Germination took place within a week of incubation in the dark at 18°C.

### Root Chamber Cultivation System (Rhizotron)

Sunflower achenes were moistened for 1–2 days, peeled, and grown on wet filter paper at 25°C. Seedlings with a root length of 2–3 cm were placed in the root chamber between a lid and filter paper as recently described (Krupp et al., 2019). The root chambers were made out of Petri dishes (9-cm diameter) filled with perlite and covered with wet filter paper (both heat-sterilized at 150°C for 2 h) with 1-cm wide holes cut into the upper side of the lid and bottom (Figure 1). The lids were fixed with adhesive tape, and the Petri dishes were placed vertically in a plastic box. Each chamber was irrigated individually, so the perlite could stay moist but without excess water in the box (the latter is important because *O. cumana* seeds are very sensitive to anaerobic conditions). The sunflower seedlings were cultivated for 5 days in a climate chamber at 22°C and daily 14-h light until sufficient roots had developed. Conditioned seeds of *O. cumana* were placed on wet filter paper at a distance of 1–2 mm to sunflower roots, but with no specific orientation of the micropyle. Cultivation continued for additional 7 days before the germination ratio and germ tube development were documented photographically under a dissecting microscope (Olympus SZ60, Olympus Corporation, Tokyo, Japan; Canon PowerShot A640, Canon, Tokyo, Japan).

### Chemotropism Bioassay

**FIGURE 2:**
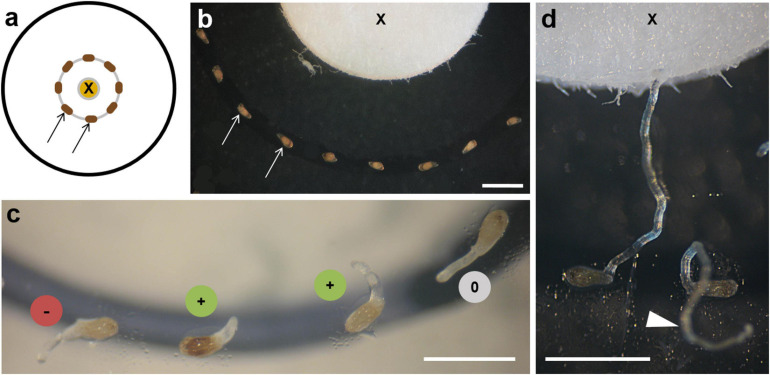
Chemotropism bioassay. **(a)** Diagram of bioassay showing the source of the test substance on the filter disc (*X*) and seeds (*arrows*) placed at a 2-mm distance. **(b)** Seeds of *O. cumana* (*arrows*) around the filter (*X*) with their micropyle facing alternately left and right. **(c)** Early stages of germ tube development: (+) bending toward the substance source; (–) away from it; (0) behaving indifferently. **(d)** Two examples of late stages of germ tube development: one touching the filter with the tested substance (*X*), the other growing without orientation above the substrate (*arrowhead*). Scale bars = 1 mm.

The chemotropism bioassay was performed in small Petri dishes (3.8-cm diameter) containing 1% water agar (Agar Agar Kobe I, Roth, Karlsruhe, Germany). A heat-sterilized 5-mm filter disc was immersed with 20 μl of the solved test substance, dried after application of the solvent, and placed in the middle of the Petri dish (Figure 2a). Under a dissecting microscope, the conditioned *O. cumana* seeds (see above) were placed at a 90° angle to the filter surface with their micropyle alternatively pointing to the left and right in a concentric circle with a 2-mm distance from the filter (Figure 2b). To start the experiment, 10 μl of ddH_2_O was pipetted onto the discs to allow dissolution and diffusion of the test substances. In tests with costunolide at concentrations of 10^–6^ M and less, the number of germinated seeds at a distance of 2 mm became too low for the observation and evaluation of a statistically relevant number of germ tubes. Therefore, 10 μl of GR24 (1 ppm) dissolved in water was used instead of ddH_2_O to ensure sufficient germination in these samples. The Petri dishes were sealed with Parafilm and kept in the dark at 18°C. After 5–7 days, germ tube curvature was recorded under a dissecting microscope. Germ tubes bending toward the tested substance were recorded as positively chemotropic (+), germ tubes bending away from it as negatively chemotropic (–), and germ tubes with their tip not showing a clear direction as indeterminable (0, Figure 2c). Among the indeterminable reactions, curling germ tubes growing upward and away from the agar were often found suggesting the influence of the orientation of the Petri dishes (Figure 2d arrowhead). However, broomrape germ tubes did not show gravitropism, as previously reported (Joel and Losner-Goshen, 1994; Fernández-Aparicio et al., 2016) and the additional tests with inversely incubated plates gave no hints for the negative gravitropic reaction of *O. cumana* germ tubes (Supplementary Figure 1).

Additional experiments to test the distance dependency of the chemotropic effect were conducted (a) with a second concentric circle with a 4-mm distance to the edge of the filter plate; (b) with seeds evenly spread without distinctly positioned micropyles with a distance from 0–2 and 2–5 mm from the tested substance.

The samples tested in the chemotropism bioassays were sunflower root exudate, a methanol extract of sunflower seed oil, costunolide, and GR24. The root exudate and seed oil extract were chosen because they were known to contain four STLs with germination-inducing capacity for *O. cumana* (Raupp and Spring, 2013) and plant growth-influencing bioactivity (Spring et al., 2020). Costunolide was selected as a model compound of the four STLs because of its commercial availability in sufficient amounts for the experiments, whereas 8-epixanthatin and tomentosin would have required laborious cultivation of suitable source plants (e.g., *Xanthium strumarium* L.), compound extraction, and purification. All the four STLs shared the structural similarity of an α,β-unsaturated-γ-lactone moiety as a bioactive center. GR24, a commercially available SL with a known germination-inducing capacity on *O. cumana* (Joel et al., 2011; Ueno et al., 2014) was used as a control to test the potential growth-influencing activity of SLs on sunflower broomrape.

### Root Exudate Collection

Sunflower plants were grown hydroponically, as described by Raupp and Spring (2013). Root exudates of 25 plants (4 weeks old) were collected by pumping the water in which they were growing over a column filled with 5 g of ion-exchange resin Amberlite (XD4, 20–60 mesh, matrix: styrene-divinylbenzene, 100 Å mean pore size, Sigma Aldrich, St. Louis, MI, United States) for 24 h at a flow rate of 35 ml/100 s. The exudate was washed from Amberlite with acetone, dried, and dissolved in 1.5 ml acetone. The roots were then dissected and weighed. The amount of exudate applied in the germination and chemotropism bioassay corresponded to the metabolites exuded with 1 g root fresh weight within 24 h.

### Sunflower Oil Extraction

Native, organic, and cold-pressed sunflower oil (Bio Planète, oil mill Moog, Lommatzsch, Germany), which had recently been used for a study on STLs in sunflower oil (Spring, 2021), was extracted 1:1 (V:V) with methanol in an Eppendorf tube, shaken, and kept for ca. 1 h for phase separation. The methanol fraction was collected with a pipette, and 20 μl of the methanol extract was applied in the bioassay. The concentration of the four STLs in the methanol extract, as determined by high pressure liquid chromatography coupled with two-dimensional mass spectrometry (HPLC–MS/MS), was: 1.08 μmol/L costunolide, 0.88 μmol/L dehydrocostus lactone, 2.16 μmol/L 8-epixanthatin, and 3.28 μmol/L tomentosin (Spring, 2021).

### Other Tested Substances

Synthetic strigolactone GR24 (C_17_H_14_O_5_) was purchased from B. Zwanenburg, Department of Organic Chemistry, Radboud University Nijmegen, Nijmegen, The Netherlands. GR24 was used at a concentration of 1 ppm (= 3.3 × 10^–6^ M) in an aqueous solution. Costunolide (C_15_H_20_O_2_) was purchased from Selleck Chemicals (Houston, TX, United States), dissolved, and diluted in methanol.

### Data Collection and Statistics

The germination ratio was calculated as germinated seeds per total number of tested seeds. At least three biological replications were examined per tested substance.

Chemotropism effects were calculated as the ratio of germ tubes showing a detectable chemotropic response (positive and negative). In about one-third of the germinated seeds, no curving direction of the germ tube could be defined either because of continuous straight growth or, more often, because curling of the germ tube caused seed movement and loss of contact to the agar surface; thus, chemical signals could no longer act (see Figure 2d, arrowhead). These cases were excluded from the evaluation (0-group). Three or more biological replications were examined per tested substance.

Values were statistically treated (mean ± standard deviation) and tested for significant differences by ANOVA (InfoStat, version 2016e, InfoStat Group, University of Córdoba, Argentina). Effects were considered significant if *p* < 0.05 in the Tukey test. Graphs were prepared with the SigmaPlot12.5 (Systat Software Inc., San Jose, CA, United States).

## Results

Rhizotron experiments (*n* = 7) with conditioned seeds of *O. cumana* showed high rates of about 50% germination when seeds were located 2 mm or less from the sunflower root. Because of root growth, overlap, and seed displacement, clear germ tube orientation could be recorded for only about half of the germinated seeds. In these cases (*n* = 193 seeds in seven independent experiments), 75 ± 5% showed curving toward the host roots, whereas 25% grew in the opposite direction (Supplementary Table 1).

To test the chemotropic influence of exudates, extracts, or candidate compounds, a bioassay was designed, which allowed the observation of the *O. cumana* seeds and germ tube development (Figure 2). The conditioned seeds were placed at a 2-mm distance around a filter disc containing the test substance, which could diffuse into the agar (Figures 2a,b). After 3–5 days of incubation at 18°C in the dark, the seeds started to germinate, and the direction of the germ tube tip was recorded photographically. The hyaline germ tubes of *O. cumana* had a conical tip that became more round over time and appeared often more opaque under the dissecting microscope (Figure 2c). Three categories of reaction were recorded: (+) for germ tubes bending toward the substance source, (–) if bending away from the source, and (0) if no distinct reaction could be defined (Figures 2c,d). The germ tubes measured approximately 100 μm in diameter and could grow up to 2–3 mm in length (Figure 2d).

**FIGURE 3:**
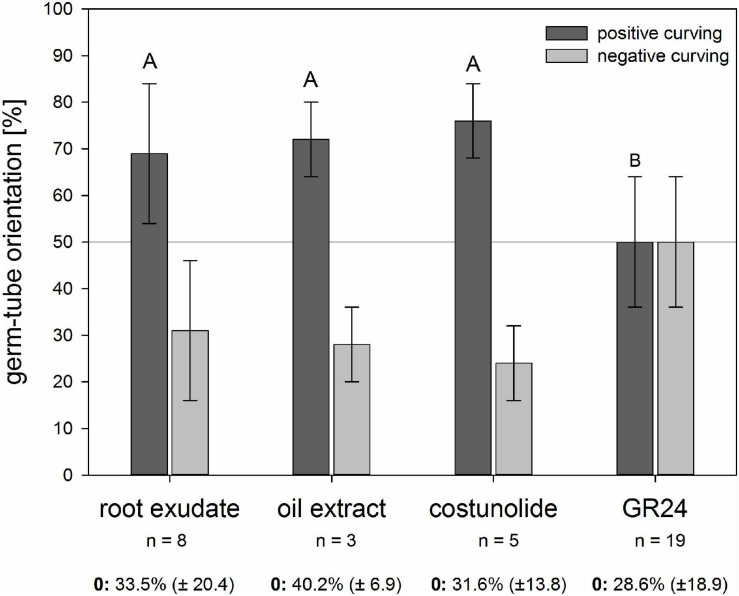
Reaction of *O. cumana* germ tubes on sunflower root exudate, sunflower oil extract, costunolide (23 ppm, 10^–4^ M), and GR24 (1 ppm, 3.3 × 10^–6^ M) as substance sources. Percentage ± standard deviation (*n* = 3–19 biological experiments) of positive (*dark gray*) and negative (*light gray*) reacting germ tubes are presented in the histogram. Germ tubes without clear orientation were not included in the calculation of the positive vs. negative reactions, but the percentage ± standard deviation of this “zero group” (0) is given below the histogram of each sample. Experiments with STLs containing samples of sunflower root exudate, sunflower oil extract, and costunolide resulted in significantly higher percentages of positive than the negative reactions compared with GR24 (Tukey test, *p* < 0.05, same letter indicates that differences are not statistically significant).

Similar to the rhizotron experiments with sunflower roots, high values of growth toward the source of chemical signals were found in chemotropism bioassays (Figure 3). All the tested samples induced seed germination, and around 70% of the oriented germ tubes showed a positive chemotropic reaction to the sunflower root exudate and sunflower oil extracts, both known to contain micromolar concentrations of STLs (Raupp and Spring, 2013; Spring, 2021). A similar positive effect (68%) was achieved when pure costunolide (23 ppm) was applied to the filter. In contrast, the strigolactone GR24 (1 ppm) led to an equal ratio of germ tubes curving toward or away from the source, which means that all the three samples containing STLs led to a significantly higher ratio of positive curving in comparison with GR24.

**FIGURE 4:**
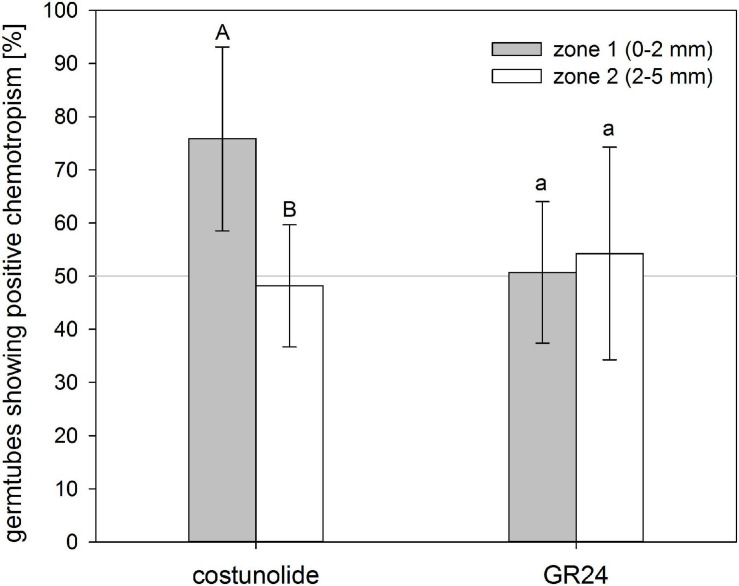
Distance-depending reaction of *O. cumana* germ tubes on costunolide. Germ tubes of seeds placed closer to the substance source showed significantly higher chemotropism percentages toward costunolide (10^–4^ M) but not toward GR24 (3.3 × 10^–6^ M). Means ± standard deviation of *n* = 5–7 replications. Same letter indicates that differences are not statistically significant (Tukey test, *p* < 0.05).

The ability to induce positive chemotropism depended on the distance from the substance source, thus indicating a concentration–activity relationship. Within a distance of 2 mm, 76 ± 17% of the germ tubes grew toward costunolide (10^–4^ M applied on the filter paper), while in the outer zone (2–5 mm), only 48 ± 11% showed positive chemotropism (Figure 4). This indicated that, because of the dilution effect with increasing distance, the concentration of the STL in the germ tube fell below the minimal active value. For GR24 (3.3 × 10^–6^ M applied on the filter paper), there was no significant difference in the ratio of positive and negative bending visible in both distances.

**FIGURE 5:**
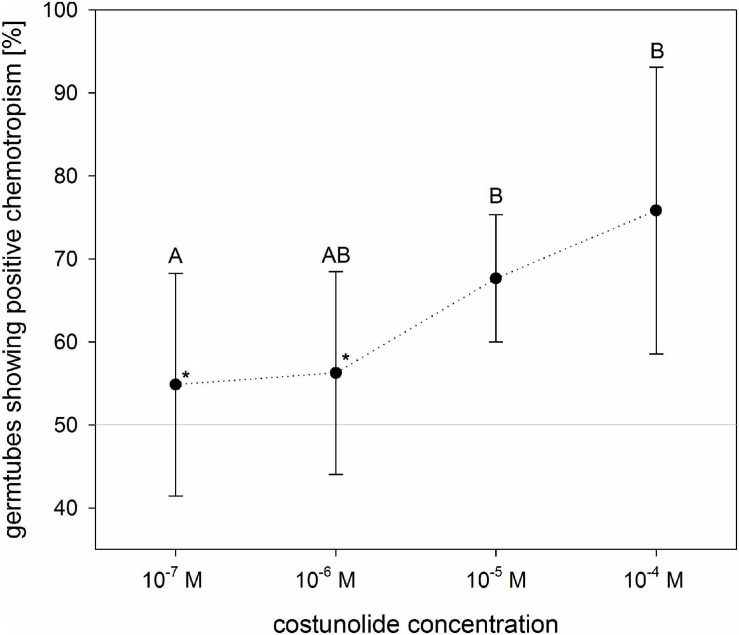
Concentration dependence of *O. cumana* chemotropism toward costunolide. The values indicate concentrations applied to the filter discs (active concentrations at the seed surface are lower but undetermined). In tests with costunolide concentrations 10^–6^ M and 10^–7^ M, GR24 was added to ensure germination (*asterisk*). Means ± standard deviation of *n* = 3–6 replications. Same letter indicates that differences are not statistically significant (Tukey test, *p* < 0.05).

To evaluate the dose dependence of costunolide on the chemotropic reaction of *O. cumana* germ tubes, different concentrations of the STL were applied on the filter discs (Figure 5). At concentrations of 10^–7^ and 10^–6^ M, the ratio of germ tubes showing positive and negative curving was nearly identical (55 ± 13% and 56 ± 12%, respectively). Positive reactions became evident at concentrations of 10^–5^ (68 ± 8%) and 10^–4^ M (76 ± 17%) applied on the filter disk. It has to be emphasized that the concentration of active compounds on the germ tube surface depends on the compound-specific diffusion constant in the given matrix, time of diffusion, and distance from the source. This cannot be determined in such an experiment without an enormous analytical effort. However, the result obtained here indicates that the minimal active concentration of costunolide for chemotropic reaction is clearly below 10^–5^ M.

## Discussion

Root parasitic plants have evolved amazing mechanisms to improve their chances of survival. Chemical signals triggering the precise timing of their seed germination and guiding the germ tube to the host surface are the first and decisive steps in their life cycle (Fernández-Aparicio et al., 2016). For germination, numerous responsible stimulants have been identified within the past 50 years since the discovery of strigolactones (Cook et al., 1966), but, intriguingly, compounds inducing a chemotropic reaction still wait to be discovered.

Results from the root chamber experiments with *O. cumana* and sunflower in this study confirmed observations from many other parasite–host combinations that seeds in close distance to the host surface show active curving of germ tubes toward the host root surface. The distance of the active reaction depends on the growth ability of the germ tube, which, for *Orobanche* spp., is usually limited to a few millimeters (Joel et al., 2007). A similar reaction distance of 2–3 mm was also observed for *O. cumana* in this study and coincided with the distance in which seed germination occurred (data not shown). This suggested that the compounds from sunflower roots responsible for the induction of both processes have identical or, at least, similar chemical characteristics in terms of water solubility, diffusion behavior, and metabolic stability. A double functionality as germination inducer and chemotropic signal had previously been assumed for strigol-like metabolites of cowpea [*Vigna unguiculata* (L.) WALP.] by Dubé and Olivier (2001), who postulated that a gradient of the compounds could serve as a signal of orientation for the host finding of *Striga gesnerioides* (WILLD.) VATKE.

The compounds to be considered as stimulators for both processes in *O. cumana* are the four STLs, which occur in similar micromolar concentrations as in sunflower roots (Raupp and Spring, 2013), cotyledons of young seedlings (Spring et al., 2020), and seed oil (Spring, 2021). They can be exudated from the rhizodermis or leached from wounded cotyledons during germination. As root chamber experiments are inappropriate for testing isolated extracts or pure compounds, we established a chemotropism assay in Petri dishes similar to that previously described by Whitney and Carsten (1981). In these chemotropism assays, sunflower root exudates and oil extracts revealed chemotropic effects (ca. 70% positive vs. 30% negative curvature) similar to those observed before in root chamber experiments. This supported the assumption that, indeed, the four STLs known as natural germination stimulants for *O. cumana* could also be responsible for the curving of the germ tubes. The tests with costunolide, selected as the model substance because of its commercial availability, confirmed its function as a chemotropic signal at concentrations above 10^–6^ M (at the site of application). In such concentrations, costunolide and the three other STLs had previously been shown to occur in sunflower roots and root exudates (Raupp and Spring, 2013), cotyledons (Spring et al., 2020), and sunflower seed oil extracts (Spring, 2021). GR24, an effective stimulant of seed germination in many *Orobanche* spp. (Yoneyama et al., 2011; Ueno et al., 2014), was used as a model to test whether strigolactones could also be involved in the germ tube curving observed in the root chamber and exudate bioassays. However, GR24 showed no chemotropic effect (nearly equal positive vs. negative curvature) at a concentration of 1 ppm, which is usually applied in germination bioassays. Strigolactones such as 5-deoxystrigol (Yoneyama et al., 2011) and heliolactone (Ueno et al., 2014) were occasionally reported from sunflower exudates when plants were cultivated under nutrient-deficient conditions, but were not found in other cases (Joel et al., 2011; Raupp and Spring, 2013). The lack of a germ tube reaction to GR24 also suggests that the observed chemotropic effect with root exudate and oil extract is due to STLs rather than SLs.

The germ tube development of *Orobanche* spp. is driven by cell elongation rather than cell division (Joel and Bar, 2013). Sunflower STLs have previously been identified as inhibitors of auxin-derived elongation growth (Spring and Hager, 1982). Hence, it appears possible that a gradient in concentration of STLs between the host-facing and the opposite flank of the *O. cumana* germ tube could interfere differently with cell elongation, thus causing curvature toward the host. Such a gradient was shown to occur in blue light-stimulated sunflower hypocotyls for 8-epixanthatin (another STL from sunflower root exudates) and was suggested to be responsible for phototropic curvature (Yokotani-Tomita et al., 1999). Moreover, the unilateral application of costunolide on sunflower hypocotyl surface was also shown to cause growth inhibition on a treated flank leading to curvature, and seemed to be a general feature of the bioactivity of α,β-unsaturated-γ-lactones (Spring et al., 2020).

The molecular mechanism of plant growth inhibition with STLs is unclear. Apart from direct inactivation of enzymes essential for the growth process (e.g., H^+^-ATPase; Hager, 2003), indirect influence *via* inhibition of the auxin-transport (Ueda et al., 2013) or regulation of gene expression (Toda et al., 2019) could be involved. More recently, an additional possibility came to light when protein modeling revealed that the two xanthanolides (8-epixanthatin and tomentosin) from sunflowers show high affinity for the KARRIKIN-INSENSITIVE2 (KAI2) hydrolases of the plant (Rahimi and Bouwmeester, 2021). KAI2 signaling is known to be involved in germination and seedling development in *Arabidopsis thaliana* (L.) Heynh. (Nelson et al., 2010) and can be activated by strigolactones. The principle is evolutionary, old, and widespread in plants (Waters et al., 2017). In Orobanchaceae, it seems to play a particular role in host recognition and the evolution of several paralogs of *KAI2*, which may be responsible for host specificity (Conn et al., 2015). It has been suggested that *D14*, a homolog of *KAI2*, could be involved in seed germination, while KAI2 proteins have a different function in parasitism (Xiong et al., 2016). Such a function could be in chemotropism. The perception of germination stimuli from the rhizosphere in *Orobanche* was suggested to be located in perisperm cells close to the micropyle of the seed (Plakhine et al., 2012), and experiments with a fluorescent ligand showed that the strigolactone receptor of *Striga hermonthica* (DELILE) BENTH. is placed in the tip cells of the germ tube (Tsuchiya et al., 2015). In *O. cumana*, the cellular situation of the embryo is similar, but the perception of STLs as natural stimulants (in addition to SLs) for the adaptation to sunflower and speciation could have required gene duplication and evolution of a new receptor, as suggested by Joel et al. (2011).

## Conclusion

The chemotropism bioassays provided evidence that STLs in exudates and extracts of sunflower not only stimulate germination of *O. cumana* seeds, as shown in previous studies (Joel et al., 2011; Raupp and Spring, 2013), but also guide the germ tube of the parasite to the host root. Costunolide, one of the four identified internal sunflower STLs used here as a model substance, induced germ tube curving at concentrations in which it occurs in the sunflower root exudate. It is most likely that the other three STLs (dehydrocostuslactone, tomentosin, and 8-epixanthatin) additionally contribute to the effect in a similar way, although this has not been proven yet. Strigolactones do not seem to play this role in the sunflower–broomrape interaction, but in other host-parasite systems where STLs do not occur, they could have a similar chemotropic function. In this context, it could be of particular interest to see the chemotropic reaction of a recently detected new race of sunflower broomrape (Dor et al., 2020), which also attacks Solanaceae, for which STLs have not been reported.

## Data Availability Statement

The original contributions presented in the study are included in the article/Supplementary Material, further inquiries can be directed to the corresponding author/s.

## Author Contributions

AK generated and analyzed the data, designed the figures, and contributed to writing and editing. BB generated and analyzed the data. OS designed the project idea, provided financial support and resources, and wrote the manuscript. All authors contributed to the article and approved the submitted version.

## Conflict of Interest

The authors declare that the research was conducted in the absence of any commercial or financial relationships that could be construed as a potential conflict of interest.

## Publisher’s Note

All claims expressed in this article are solely those of the authors and do not necessarily represent those of their affiliated organizations, or those of the publisher, the editors and the reviewers. Any product that may be evaluated in this article, or claim that may be made by its manufacturer, is not guaranteed or endorsed by the publisher.

## Supplementary Material

Supplementary Figure 1 Orientation of *O. cumana* germ tubes in relation to gravity. Percentage of germ tubes growing away from the substrate if the Petri dish was either placed bottom-down (*n* = 5 per substance) or bottom-up (*n* = 2 per substance). In both cases, and with both STLs costunolide (10^–4^ M) and strigolactone GR24 (3.3 × 10^–6^ M), around 60% of the germ tubes grew away from the substrate and showed no gravitropism. Percentages are means ± standard deviation. No significant differences between the two treatments were found by the Tukey test (*p* < 0.05).

Supplementary Table 1 Chemotropism data from root chambers and chemotropism bioassays.
